# Characteristics of exopolysaccharides produced by isolates from natural bioflocculant of *Ruditapes philippinarum* conglutination mud

**DOI:** 10.3389/fmicb.2022.1068922

**Published:** 2023-01-11

**Authors:** Lijuan Feng, Tingting Qian, Guangfeng Yang, Jun Mu

**Affiliations:** ^1^Zhejiang Provincial Key Laboratory of Petrochemical Pollution Control, Zhejiang Ocean University, Zhoushan, Zhejiang, China; ^2^School of Ecology and Environment, Hainan Tropical Ocean University, Sanya, Hainan, China

**Keywords:** exopolysaccharides, *Ruditapes philippinarum* conglutination mud, biological activities, flocculation, decolorization, antioxidation

## Abstract

Three novel types of exopolysaccharides (EPS) EPS-S8, EPS-S5, and EPS-F10 were extracted and purified from bacterial isolates *Bacillus* sp. GHS8, *Pseudoalteromonas* sp. GHS5 and *Psychrobacter* sp. GHF10, which were originated from natural bioflocculant of *Ruditapes philippinarum* conglutination mud (RPM), respectively. The EPS had similar function groups C-H, N-H, C-O, and C = O. The EPS were composed of different monosaccharides (EPS-F10, Man: GlcN: GlcUA: GalUA = 1:0.66:5.75:0.51; EPS-S5, Man: Gal: GlcN: Rib = 1: 0.50: 2.94: 0.26; EPS-S8, Man: Gal: GlcN = 1:1.54:7.69). The molecular weights (Mw) of EPS were ordered as 51.4 kDa (EPS-S5) > 9.15 kDa (EPS-S8) > 4.41 kDa (EPS-F10). Three types of EPS all showed higher peak flocculation activities than the reported crude EPS from the RPM. Besides, the EPS also exhibited efficient decoloration and antioxidation activities, especially for EPS-S8, which might be due to the low Mw and specific monosaccharide composition.

## 1. Introduction

Exopolysaccharides (EPSs) are extracellular metabolites of living organisms (plants, animals, algae, bacteria, and fungi) (Osemwegie et al., 2020; Cheong et al., 2022; Garza-Rodríguez et al., 2022). In the process of growth and metabolism, some specific bacteria can secrete a class of extracellular polysaccharides, some of which adhere to the cell wall to form capsules, and some of which enter the medium to form mucus, both of them are called EPS (Díaz-Cornejoa et al., 2023). EPS has been widely studied over the past decades due to its extensive sources, easy cultivation and diverse biological functions, such as immunomodulation, antioxidative, antitumor, antimicrobial, emulsification, antiradiation, etc. (Hua et al., 2010; Yildiz and Karatas, 2018; Prateeksha et al., 2021; Sindhu et al., 2021). The vast sea area is a treasure source of marine microbial resources. Compared with terrestrial microorganisms, the EPS produced by marine microorganisms have different biological activities and structural characteristics because of the high-pressure and high-salt marine natural environment (Yan et al., 2016; Padmanaban et al., 2022). Some researchers have isolated bacteria which produce various EPS from different marine circumstances, such as *Bacillus licheniformis* (Arena et al., 2006), *Bacillus cereus* (Peele et al., 2016), *Edwardsiella tarda* (Guo et al., 2010), *Sphingobium yanoikuyae* BBL01 (Kant Bhatia et al., 2021), *Acinetobacter* (Peele et al., 2016), *Pseudoalteromonas* (Roca et al., 2016)*, Bacillus enclensis* AP-4 (Hu et al., 2022). The chemical structure of EPS plays important roles in the biological functions. The activity of EPS was considered to be related to the molecular weight (Mw), anomeric configuration, and monosaccharide composition (Guo et al., 2010; Joulak et al., 2020; Vinothkanna et al., 2021). However, the microbial isolates from marine environment are still not enough, and the biological function with the structure of EPS needs further study.

*Ruditapes philippinarum*, a very popular mudflat shellfish, is widely distributed in the coast and received much attention (Bi et al., 2022; Marisa et al., 2022). In our previous studies, we firstly revealed EPS as a novel natural bioflocculant resource from *Ruditapes philippinarum* conglutination mud (RPM), and two complex heteropolysaccharides (Mw, 5.7 and 18.0 kDa) were screened with similar monosaccharides composition except glucose content (Mu et al., 2018). The RPM contained abundant bacteria (Mu et al., 2019b), and 14 bacterial strains were further isolated from the Zhoushan RPM, including *Pseudoalteromonas* sp., *Psychrobacter* sp., *Halomonas* sp., *Albirhodobacter* sp., *Celeribacter* sp., *Kocuria* sp., and *Bacillus* sp. The crude EPS of these isolated bacterial strains were proved to have efficient bioflocculation (Mu et al., 2019a). Thus, the bacteria in RPM plays important roles in the bioflocculation. However, the pure active polysaccharides of the flocculating bacteria in RPM have never been isolated and characterized, and the full composition characterization of purified EPS from the isolates needs further elucidation for practical purposes. We hypothesized that novel purified EPS with efficient bioflocculation could be obtained from the isolates from RPM. Besides, more function of these new types of EPS from isolates of RPM should be also studied because of the potential diverse biological function of the bacterial EPS.

In the present study, three flocculating bacterial strains were obtained from the RPM, and different EPS from these isolates were purified. The aims of this study are to (1) purify novel EPS and analyze their structural characterization, including functional groups and monosaccharides components; (2) study the diverse biological functions of the different EPS, including flocculation, decolorization and antioxidation.

## 2. Materials and methods

### 2.1. Extraction of EPS

Three bacterial strains *Psychrobacter* sp. GHF10, *Pseudoalteromonas* sp. GHS5, and *Bacillus* sp. GHS8 isolated from the RPM, were identified by the 16S rDNA sequences analysis with the NCBI accession Numbers of KX702266, KX702256, and KX702261, respectively. Each bacterial strain was inoculated in a 10 L fermentation tank (BIOTECH-7BG-3, Baoxing, China) with culture medium. The compositions of medium were presented as followings (g L^−1^): glucose, 20; (NH_4_)_2_SO_4_, 0.2; urea, 0.5; yeast extract, 0.5; MgSO_4_·7H_2_O, 0.2; KH_2_PO_4_, 2.0; K_2_HPO_4_, 5.0. The components were dissolved in synthetic seawater, which was composed of the followings substances (g L^−1^): MgCl_2_·6H_2_O, 9.68; KCl, 0.61; Na_2_SO_4_, 3.47; NaCl, 30.0; Na_2_HPO_4_, 0.014; NaHCO_3_, 0.17; CaCl_2_·2H_2_O, 1.36; KBr, 0.10; SrCl_2_·6H_2_O, 0.04; H_3_BO_3_, 0.03. After culture for 48 h in the condition of 25°C and 180 rpm, 6 L of fermentation broth was obtained and centrifuged at 9,000 *g* for 20 min, and the supernatant was preserved and concentrated. The concentrated solution was packed into a dialysis bag with a Mw cut-off of 3,500 Da. The dialysis bag was placed in deionized water and dialyzed for 2 days at 4°C to remove salts and other small molecular compounds. The deionized water was replaced every 120 min. Then, the dialysate was concentrated by rotary evaporator (RE-2000). Finally, the concentrated solution was mixed with threefold of cold ethanol at 4°C for 1 day, and the precipitation was crude EPS. All the materials were used under aseptic conditions.

The Sevag method was utilized to remove protein in the crude EPS (Staub, 1965). The total polysaccharides of crude EPS was determined by the phenol-sulfuric acid method (DuBois et al., 1956). The EPS purity was identified by ultraviolet spectroscopy (UV) scanning at a wavelength range of 200–400 nm. The purification was further proceeded by an anion exchange chromatography (DEAE-52 cellulose column) and gel permeation chromatography (Sephadex G-100 column). The gradient concentrations of NaCl solutions (0, 0.1, 0.3, and 0.5 mol L^−1^) were used as the eluent to elute the anion exchange column gradually with a flow rate of 1 ml min^−1^. Thus, three types of pure EPS were obtained and named as EPS-F10 (*Psychrobacter* sp. GHF10), EPS-S5 (*Pseudoalteromonas* sp. GHS5), and EPS-S8 (*Bacillus* sp. GHS8), respectively.

### 2.2. Structure analysis of purified EPS

The Mw of the purified EPS was determined by high performance gel permeation chromatography (HP-GPC) coupled with a TSK gel G3000PWXL column (7.8 mm × 30.0 cm, Tosoh, Japan) and the refractive index detector (Wang et al., 2020). The detection conditions were presented as follows: the mobile phase was 0.1 M sodium nitrate solution; the injection volume, column temperature and flow rate were 0.02 ml, 30°C, 0.8 ml min^−1^, respectively. A satisfactory standard (*r*^2^ > 0.999) was made by different Mw (2.7–133.8 kDa) of dextran standards. Then, the Mw of the samples were calculated using the GPC software.

The hydrolysis of purified EPS was the same as previous study (Mu et al., 2019a). Then, the monosaccharide compositions of the hydrolyzed EPS were also determined by PMP pre-column derivatization HPLC (Agilent HP 1100, Agilent Technologies, United States). The chromatographic column was Agilent’s ZORBAX EclipesXDB-C18 column with a specification of 4.6 μm × 250 mm 5 μm; a diode array detector; column temperature, 30°C; mobile phase,17.6% acetonitrile: 82.4% phosphate (0.1 M, pH = 6.7); flow rate, 1 ml min^−1^; injection volume, 20 μl; detector wavelength, 245 nm. Twelve kinds of monosaccharide standards were used for drawing standard curves, including mannose (Man), galactose (Gal), xylose (Xyl), glucosamine (GlcN), ribose (Rib), rhamnose (Rha), arabinose (Ara), galacturonic acid (GlaUA), galactosamine (GalN), glucose (Glc), fucose (Fuc), and glucuronic acid (GlcUA).

The FT-IR analysis was utilized for ascertaining the function groups of purified EPS. The mixture of dried EPS (1 mg) and KBr (0.1 g) were pressed and then scanned in the frequency range of 4,000–400 cm^−1^ and speed of 1 cm^−1^.

#### 2.2.1. Flocculation activity

Flocculation activity of the purified EPS were determined using the modified method. Briefly, various concentrations of EPS (0, 0.1, 0.2, 0.4, 0.6, 0.8, 1 mg mL^−1^) were mixed with kaolin suspension (4 g L^−1^) and CaCl_2_ solution (10 g L^−1^) at the rotation speed of 180 r min^−1^ for 1 min and then mixed at 30 r min^−1^ for 2 min. The supernatant was collected after 10 min precipitation and measured at wavelength of 550 nm by an ultraviolet spectrophotometer. The calculation of the flocculation rate is shown in Eq. (1):


(1) $$\text{FA}\left(\%\right)=\frac{A_{0}-A}{A_{0}}\times 100\%$$

Where, FA, the flocculation rate of the sample; *A*_0_ and *A* are the absorbance values of the control group and the sample at 550 nm, respectively.

#### 2.2.2. Decolorization activity

Crystal violet (0.4 g mL^−1^) was used for determining the decolorization activity of purified EPS. The mixture of dyes solution was stirred with 0–1 mg ml^−1^ EPS at 30 rpm min^−1^ for 1 min. After precipitation and centrifuge, the supernatant was measured at wavelength of 620 nm. The calculation formula of decolorization activity is shown in Eq. (2):


(2) $$\text{DC}\left(\%\right)=\frac{A_{0}-A}{A_{0}}\times 100\%$$

Where, DC, the decolorization rate; *A*_0_, the absorbance value of the control sample; *A*, the absorbance value of the treatment samples.

#### 2.2.3. Antioxidant activity

DPPH radical scavenging assay was conducted in this study according to a previous study (Vinothkanna et al., 2021). Various concentrations of purified EPS were prepared, and 1 ml of which were mixed with 5 ml of 4 mM DPPH solution (dissolved in 95% ethanol), respectively. The mixture was incubated for 0.5 h under dark condition, and the absorbance was determined at wavelength of 517 nm. The DPPH radical scavenging ability was measured by Eq. (3):


(3) $$R_{\text{DPPH}}\left(\%\right)=\left(1-\frac{A_{2}-A_{1}}{A_{0}}\right)\times 100\%$$

Where, *R*_DPPH_, the DPPH clearance rate of the sample; *A*_2_, the absorbance value of the sample; *A*_1_, the absorbance value of the reference group; *A*_0_, the absorbance value of the control group.

OH radical scavenging assay was also conducted. The various concentrations of purified EPS (0.5 ml) were mixed with 1 ml H_2_O_2_ solution (6 mM) and 0.5 ml FeSO_4_ solution (9 mM), and then bathed in a water bath at 25°C for 10 min. Finally, 1 ml of 9 mmol L^−1^ salicylic acid was added in a water bath at 37°C for 1 h. The control group was distilled water. The calculation of the scavenging rate of hydroxyl radicals of polysaccharide samples is shown in Eq. (4):


(4) $$R_{\text{OH}}=\left(1-\frac{A_{1}}{A_{0}}\right)\times 100\%$$

Where, *R*_OH_, the OH clearance rate of the sample; *A*_1_, the absorbance value of the sample at wavelength of 510 nm; *A*_0_, the absorbance value of the blank group.

## 3. Results and discussion

### 3.1. Extraction of EPS

**Figure 1:**
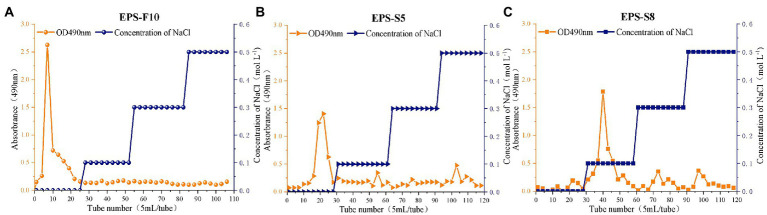
The elution curves of extracellular crude EPS-F10 **(A)**, EPS-S5 **(B)**, and EPS-S8 **(C)** by anion exchange column chromatography.

**Figure 2:**
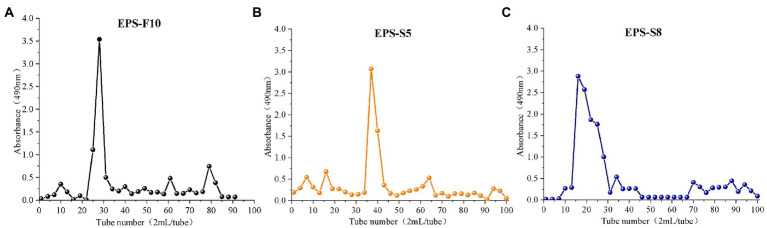
The elution curves of EPS-F10 **(A)**, EPS-S5 **(B)**, and EPS-S8 **(C)** by gel permeation chromatography column.

Three types of crude EPS (142.03, 107.03, and 111.60 mg) from the three strains (*Psychrobacter* sp. GHF10, *Pseudoalteromonas* sp. GHS5, and *Bacillus* sp. GHS8) had the percentages of total polysaccharides of 87.02, 92.16, and 78.2%, respectively. The isolated EPS were scanned under the ultraviolet spectrum (200–400 nm) and there were no sharp absorption peaks of protein or nucleic acid. The EPS were further purified *via* DEAE-52 Cellulose Ion Exchange Chromatography (Figure 1) and gel permeation chromatography Sephadex G-100 (Figure 2), and the single EPS fraction was obtained.

### 3.2. Molecular weight (Mw) and FT-IR analysis

**Figure 3:**
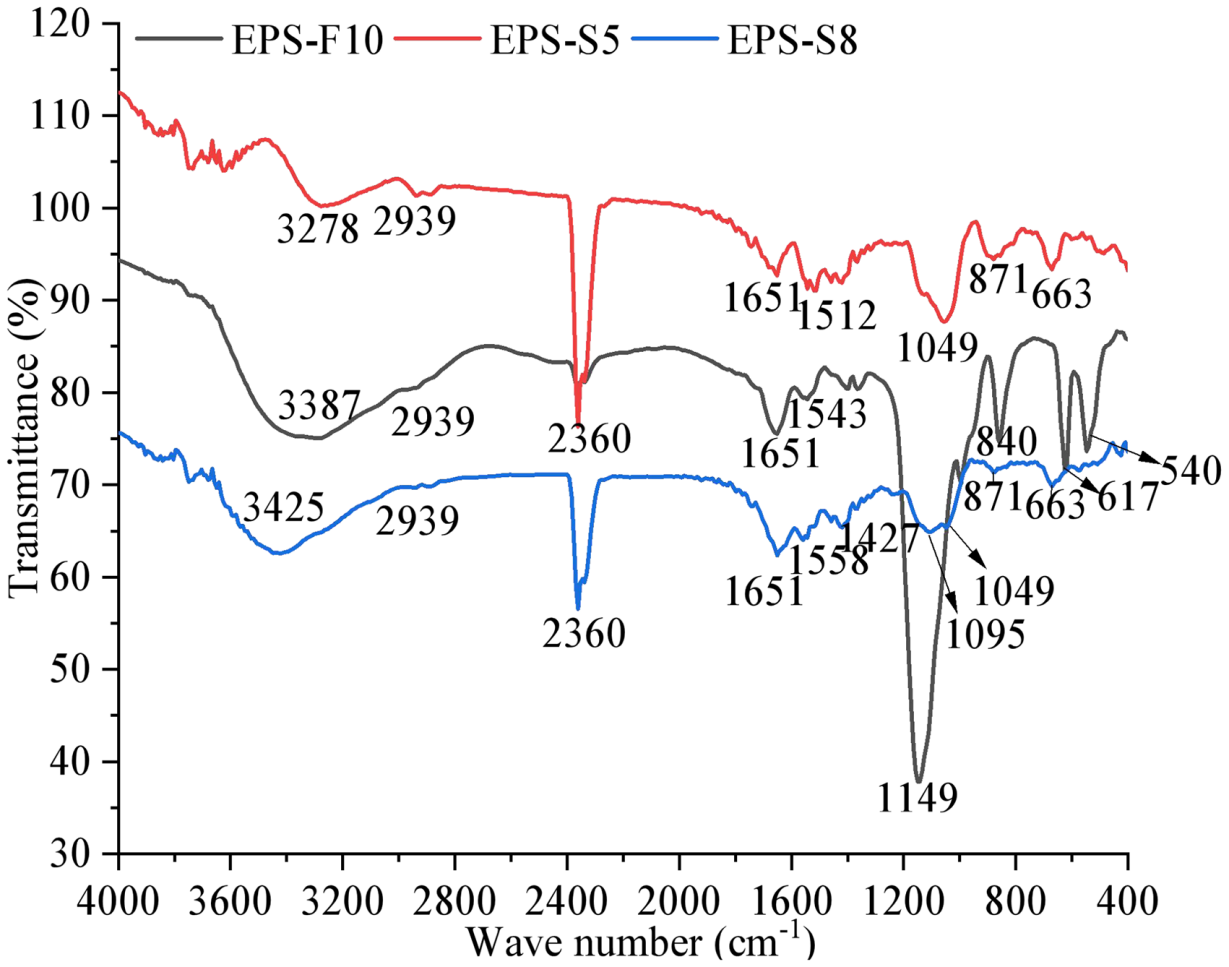
The Fourier infrared spectrum of EPS from three strains.

The Mw of the three EPS were ordered as 51.4 kDa (EPS-S5) > 9.15 kDa (EPS-S8) > 4.41 kDa (EPS-F10) (Supplementary Figure S1). Figure 3 presents function groups of pure EPS *via* FT-IR analysis. The common five spectral regions were all found in the polysaccharide IR spectrum (Hong et al., 2021). The broad absorption peak at 3,387, 3,278, and 3,425 cm^−1^, which were identified as the stretching vibration of –OH or N-H (Govindan et al., 2021; Yilmaz et al., 2022). The same peak of C-H stretching vibrations and characteristic of C=O were observed at 2,939 and 1,651 cm^−1^, respectively (Liu et al., 2020; Xia et al., 2022). Besides, the peaks at 1,149, 1,095, and 1,049 cm^−1^ were the characteristic absorption peaks of C-O single bond in polysaccharide derivatives, indicative of the presence of pyranose (Li et al., 2021). Besides, the characteristic absorption peak of α-pyranose were observed at 840 and 871 cm^−1^ (Cheng et al., 2013). Results showed that the three types of EPS all had similar function groups of –OH/N-H, C-H, C-O, C=O, the EPS-S5 and EPS-S8 were more similar than that of EPS-F10.

### 3.3. Monosaccharide composition analysis

**Figure 4:**
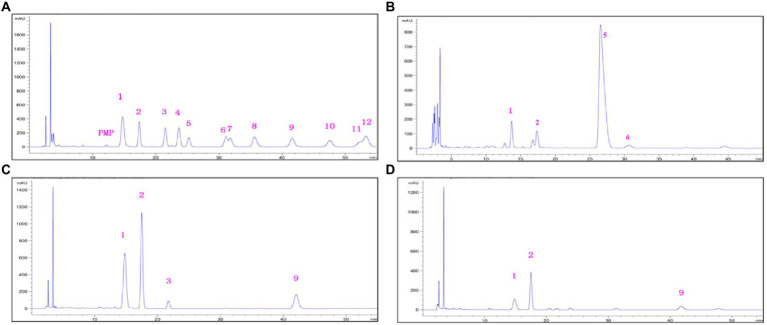
HPLC chromatograms o of standard monosaccharides **(A)**, monosaccharides derivatives from EPS-F10 **(B)**, EPS-S5 **(C)**, and EPS-S8 **(D)** (1, Man; 2, GlcN; 3, Rib; 4, Rha; 5, GlcUA; 6, GalUA; 7, GalN; 8, Glc; 9, Gal; 10, Xyl; 11, Ara; 12, Fuc).

The monosaccharide compositions of EPS are presented in Figure 4. The EPS-F10 were composed of Man, GlcN, GlcUA, and GalUA, with a composition ratio of 1:0.66:5.75:0.51. The EPS-S5 consisted of Man, Gal, GlcN, and Rib, with a ratio of 1: 0.50: 2.94: 0.26. Besides, the EPS-S8 was composed of Man, Gal, and GlcN, with the ratio of 1:1.54:7.69. Thus, three types of EPS belonging to heteropolysaccharides, consisted of three or more kinds of monosaccharide units (Man, Gal, GlcN, etc.), while the EPS-S5 merely contained Rib and EPS-F10 merely had acid sugars (GalUA, GlcUA). In addition, neutral sugars (Man, Gal, Glc, Rib) accounted for 12.6% (EPS-F10), 37.4% (EPS-S5), and 24.8% (EPS-S8). Relative abundances of amino sugars (GlcN) were 8.3, 62.6, and 75.2%, respectively. Thus, the monosaccharides of EPS-S5 and EPS-S8 belonged to neutral sugars and amino sugars, while EPS-F10 contained three types of monosaccharides (acid sugars, neutral sugars, and amino sugars). EPS-S8 had much simpler monosaccharides composition than those of EPS-S5 and EPS-F10.

### 3.4. The activity of purified EPS

**Figure 5:**
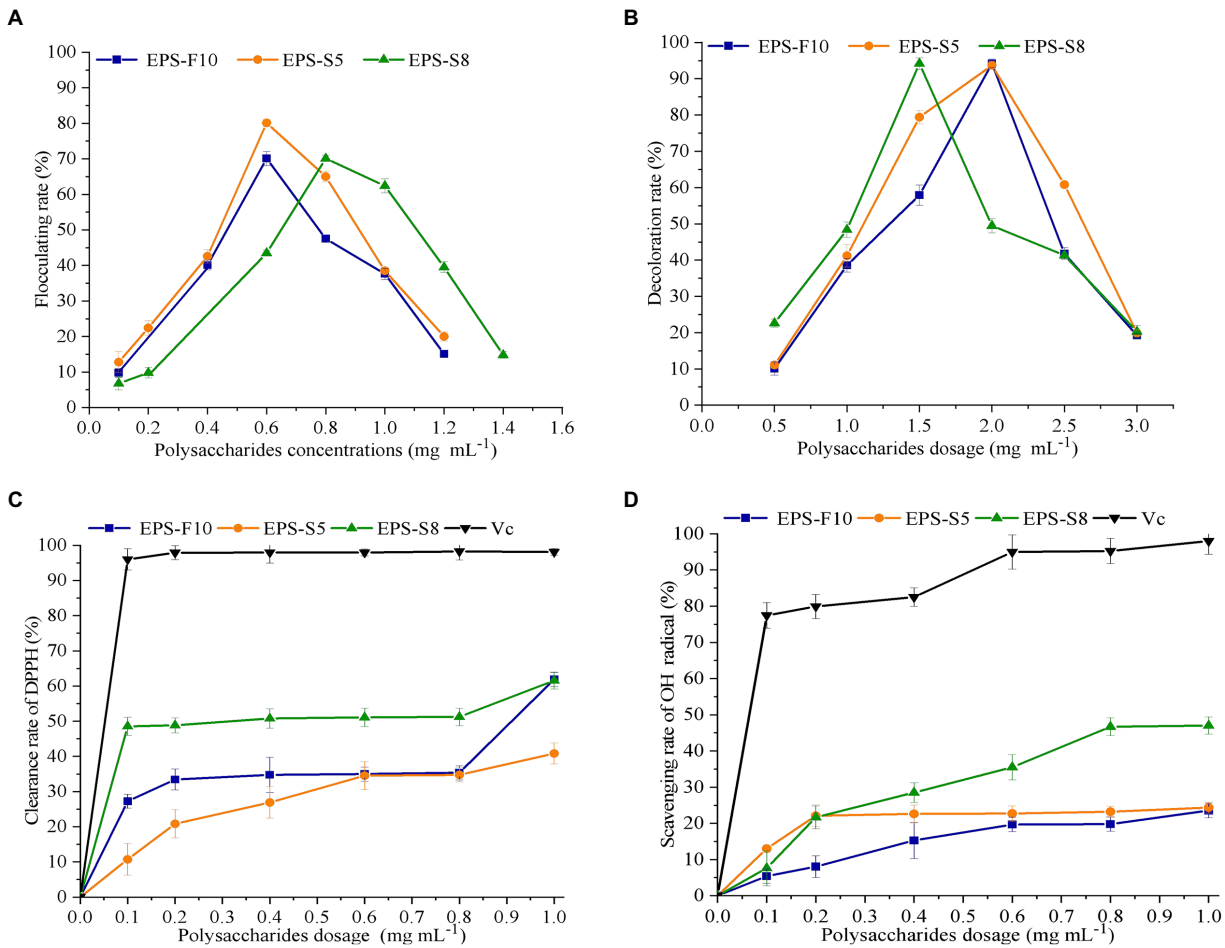
The effect of EPS dosage on flocculation rate **(A)**, decoloration rate **(B)**, scavenging activity on DPPH **(C)**, and OH **(D)**.

The flocculation activity of the different EPS was determined in kaolin clay (4 g L^−1^) and CaCl_2_ solution (10 g L^−1^) (Figure 5A). The flocculation rates of the three types of EPS demonstrated a first rise and fall trend in the range of 0.0–1.4 mg mL^−1^. The peak flocculation rates of the EPS occurred at 0.6 mg mL^−1^ (EPS-F10), 0.6 mg mL^−1^ (EPS-S5), and 0.8 mg mL^−1^ (EPS-S8), respectively. Besides, the EPS-S5 had the highest peak flocculation rates (80.1%).

In terms of decoloration activity, similar phenomenon of first rise and then fall trend were found in the three types of EPS (Figure 5B). The same maximum decoloration rates of 94.0% were observed at EPS concentrations of 2.0 mg mL^−1^ (EPS-F10), 2.0 mg mL^−1^ (EPS-S5), and 1.5 mg mL^−1^ (EPS-S8), respectively. Obviously, the EPS-S8 exhibited better performance of decoloration activity.

The scavenging ability of DPPH radicals and hydroxyl radicals were important indicators of antioxidant property. The removal of two free radicals by the three types of EPS demonstrated a concentration-dependent manner. As the concentrations of EPS increased in the range of 0–1.0 mg mL^−1^, the scavenging ability of the two free radicals gradually became stronger, but much lower than those of ascorbic acid (Vc). The DPPH radical scavenging ability reached 61.9% (EPS-F10), 40.8% (EPS-S5), and 61.6% (EPS-S8) at an EPS concentration of 1 mg mL^−1^, and the EC50 values of scavenging ability of DPPH radicals were 0.23 mg mL^−1^, 0.35 mg mL^−1^, and 0.24 mg mL^−1^, respectively (Figure 5C). Results suggested that the DPPH radical scavenging ability of EPS-F10 and EPS-S8 was significantly higher than those of EPS-S5. In addition, at a concentration of 1 mg mL^−1^, the scavenging ability of EPS on hydroxyl radicals were 23.5% (EPS-F10), 24.5% (EPS-S5), and 47.0% (EPS-S8), respectively (Figure 5D). The EPS-S8 both exhibited strong scavenging ability on DPPH and hydroxyl radicals. Thus, all pure EPS from the three strains had effective antioxidant attribute.

## 4. Discussion

There were a variety of bacteria reported to produce EPS with biological functions. In the present study, three bacterial strains isolated from RPM produced heteropolysaccharides which contained a magnitude of 10^3^−10^4^ of Mw, multi-types of monosaccharides (Man, Gal, GlcN, GalUA, etc.) and function groups (C-H, N-H, C-O, -C=O, etc.). To our knowledge, these EPS molecules showed no similarity with reported microbial bioflocculants for their specific molecular weights and complicated composition (Shahadat et al., 2017; Hu et al., 2019; Mathivanan, et al., 2020; Pu et al., 2020; Vinothkanna et al., 2021; Xia et al., 2022). The purified EPS produced in this study could be the novel EPS. Besides, these heteropolysaccharides plays roles in different fields, including flocculation, decoloration, and antioxidation.

In our previous study, two complex crude heteropolysaccharides (RPMP-1 and RPMP-2) have been extracted from RPM (Mu et al., 2018). The Mw of RPMP-1 and PRMP-2 were 5.7 and 18.0 kDa, both composed of Gal, GalN, GalUA, Glc, GlcN, GlcUA, Man, Rha, Xyl, Ara, and Fuc. Furthermore, the crude EPS of some bacterial isolates from the RPM exhibited highly similar monosaccharide composition to the RPMP-1 (Mu et al., 2019b). The purified heteropolysaccharides produced by the three bacterial strains in this study had similar magnitude of Mw and simpler monosaccharide composition than those of these crude EPS. The peak flocculation efficiency of RPMP and crude EPS of bacterial isolates were at the range of 25.4–55.0%, which was much lower than the three types of purified heteropolysaccharides (70.1–80.1%) in this study. These results proved that the three types of purified EPS demonstrated highly efficient flocculation activity.

Up to date, a variety of strains producing EPS with the function of efficient flocculation have been reported. The peak flocculation activity of these EPS in this study was achieved at the similar level of dosage with those of several reported strains, including *Leuconostoc mesenteroides* strain XR1 (Wang et al., 2021), *Streptococcus thermophilus* ZJUIDS-2-01 (Cao et al., 2021). Furthermore, the peak flocculation activity of the three types of purified EPS were much higher than those from strains *Halomonas elongata* S6 (Joulak et al., 2020), *Pseudoalteromonas* sp. SM9913. Thus, the results further proved that the purified EPS demonstrated highly efficient flocculation activity in this study. The flocculation activity demonstrated a concentration-dependent manner, and the high concentration of EPS was not favored for flocculation performance mainly due to the incompletely dispersion. There were also several strains reported to have EPS with the multi-biological activity of flocculation and antioxidation, like *Bacillus licheniformis* AG-06 (Vinothkanna et al., 2021), *Halomonas elongata* S6 (Joulak et al., 2020), *Streptococcus thermophilus* ZJUIDS-2-01 (Cao et al., 2021).

The reported EPS produced by the same genera with function of flocculation, decoloration or antioxidation as this study (*Psychrobacter*, *Pseudoalteromonas*, and *Bacillus*) is presented in Table 1. Up to date, there have been several studies reporting the biological function of EPS from genera *Pseudoalteromonas* and *Bacillus*, but this study firstly reported the EPS from genera *Psychrobacter*. Besides, there is still little information about the function of flocculation, decoloration or antioxidation from the both genera *Pseudoalteromonas* and *Psychrobacter*. In our previous study, the crude EPS of 14 strains isolated from the Zhoushan RPM were proved to have efficient bioflocculation (Mu et al., 2019a). The purified EPS of three strains have been further screened and identified in this study, and the function of flocculation, decoloration, or antioxidation were further studied. This result further suggested that the EPS produced from bacteria play important roles in the RPM, and the purified EPS exhibited the higher peak flocculation activity than the crude EPS from the RPM.

**Table 1:** The reported EPS produced by the same genera with function of flocculation, decoloration, or antioxidation as this study (*Psychrobacter*, *Pseudoalteromonas*, and *Bacillus*).

Source	Mw (kDa)	Monosaccharide composition	Biological activity			References
			Flocculation	Decoloration	Antioxidation	
***Psychrobacter* sp. GHF10**	**4.41**	**Man, GlcN, GlcUA, GalUA**	**√**	**√**	**√**	**This study**
***Pseudoalteromonas* sp. GHS5**	**51.4**	**Man, GlcN, Rib, Gal**	**√**	**√**	**√**	**This study**
*Pseudoalteromonas* sp. *SM9913*	/	/	√	nr	nr	Li et al. (2008)
***Bacillus* sp. GHS8-1**	**9.15**	**Gal, GlcN, Man**	**√**	**√**	**√**	**This study**
*Bacillus* sp. S-1	17.65	Gal, Glc, and Man	nr	nr	√	Hu et al. (2019)
*Bacillus megaterium* strain PL8	4,550	Gal, GalUA, Glc, GlcUA, and Man	√	nr	nr	Pu et al. (2020)
*Bacillus cereus* KMS3-1	/	Man, Rha, Glc, Xyl	√	nr	nr	Mathivanan et al. (2020)
*Bacillus licheniformis* AG-06	nr	Man, Gal, Rha, Xyl, Glc	√	nr	√	Vinothkanna et al. (2021)
*Bacillus subtilis* ZHX3	10.02	Rha, Ara, Gal, Glc, Man, GlaUA	√	√	nr	Xia et al. (2022)

nr, not reported.

The Mw of bacterial EPS varied between 10 and 5,000 kDa in common. Three types of EPS had lower magnitude of Mw (4.41–51.4 kDa). EPS-S5 with the highest Mw exhibited the lowest DPPH scavenging ability in this study. The low Mw polysaccharide were reported to exhibit stronger antioxidant activity than high Mw of polysaccharide (Zhang et al., 2018), which was similar in this study. The monosaccharides of EPS-S8 all belonged to neutral sugars, which had much simpler monosaccharide composition than EPS-F10 and EPS-S5. Therefore, the high antioxidant activity of EPS-S8 might be explained by the specific monosaccharide composition.

## 5. Conclusion

Three novel heteropolysaccharides with flocculation, decoloration, and antioxidation activities were generated and purified from *Bacillus* sp. GHS8, *Pseudoalteromonas* sp. GHS5 and *Psychrobacter* sp. GHF10 isolated from the RPM, respectively. Similar function groups (C-H, N-H, C-O, –C=O, etc.), the similar magnitude (4.41–51.4 kDa) of Mw, and neutral sugars were all found in the three types of EPS. However, EPS-S8 from *Bacillus* had low Mw, specific monosaccharide composition, which exhibited the better performance of decoloration activity and scavenging ability on both DPPH as well as hydroxyl radicals.

## Data availability statement

The original contributions presented in the study are included in the article/Supplementary material, further inquiries can be directed to the corresponding author.

## Author contributions

LF: conceptualization, methodology, software, data curation, visualization, writing – original draft preparation, and writing – reviewing and editing. TQ: data curation, visualization, methodology, software, and visualization. GY: visualization, methodology, software, and writing – reviewing and editing. JM: conceptualization, resources, and writing – reviewing and editing. All authors contributed to the article and approved the submitted version.

## Funding

The authors gratefully acknowledge the financial support by the Scientific Research Foundation of Hainan Tropical Ocean University (No. RHDRC202118).

## Conflict of interest

The authors declare that the research was conducted in the absence of any commercial or financial relationships that could be construed as a potential conflict of interest.

## Publisher’s note

All claims expressed in this article are solely those of the authors and do not necessarily represent those of their affiliated organizations, or those of the publisher, the editors and the reviewers. Any product that may be evaluated in this article, or claim that may be made by its manufacturer, is not guaranteed or endorsed by the publisher.

## Supplementary material

The Supplementary material for this article can be found online at: https://www.frontiersin.org/articles/10.3389/fmicb.2022.1068922/full#supplementary-material
