# Supplementary material for: Characteristics of exopolysaccharides produced by isolates from natural bioflocculant of Ruditapes philippinarum conglutination mud
Source: Front Microbiol. 2023 Jan 11;13:1068922. doi: 10.3389/fmicb.2022.1068922 (PMC9874098; doi:10.3389/fmicb.2022.1068922)
Supplement: Supplementary file 1 [file Image_1.pdf]

Supplementary data

Characteristics of exopolysaccharides produced by isolates from natural bioflocculant of *Ruditapes philippinarum* conglutination mud

Lijuan Feng[a#], Tingting Qian[a#], Guangfeng Yang[a], Jun Mu[b]*

[a]Zhejiang Provincial Key Laboratory of Petrochemical Pollution Control, Zhejiang Ocean University, Zhoushan City, Zhejiang Province, People's Republic of China

[b]School of Ecology and Environment, Hainan Tropical Ocean University, Sanya City, Hainan Province, People's Republic of China

[#] Lijuan Feng and Tingting Qian contribute equally to this paper

[*]Corresponding author: Jun Mu, mujun@zjou.edu.cn

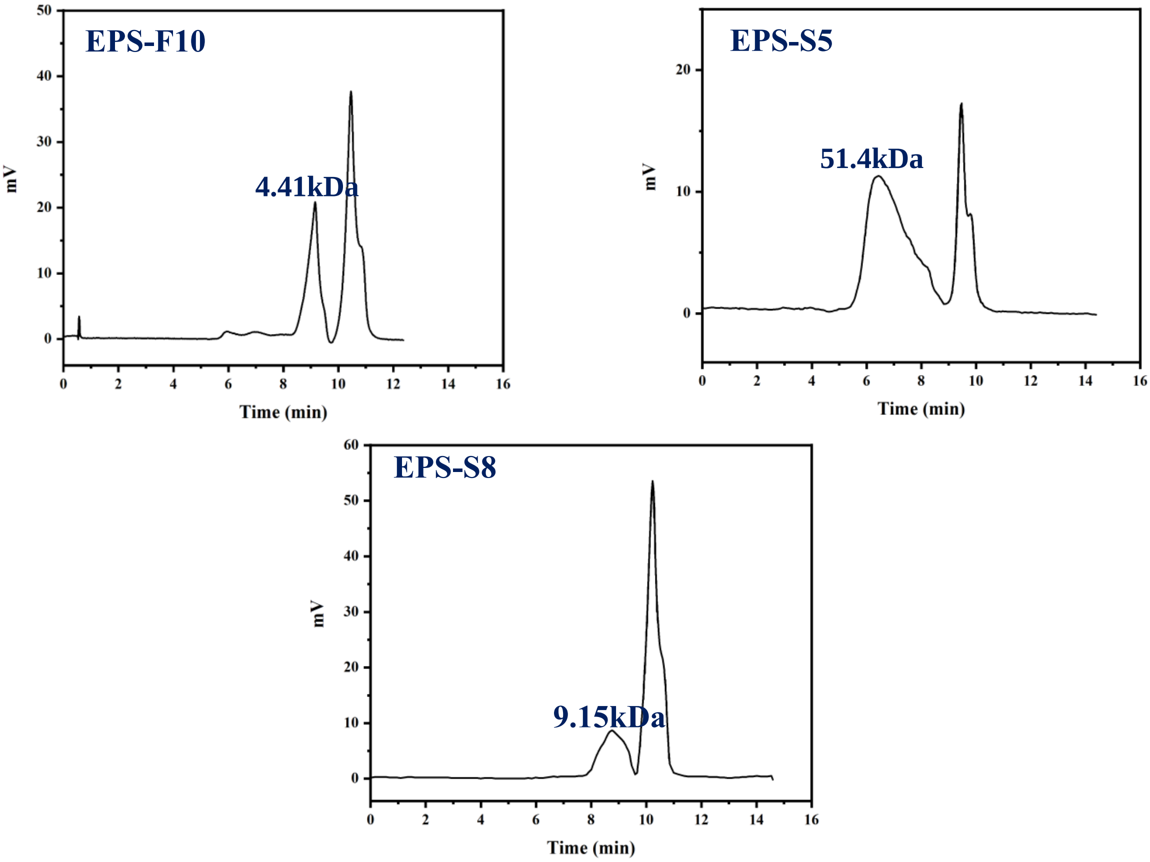

Fig. S1 The Mw of the three EPS by HP-GPC
